# Developing a comprehensive response for treatment of children under 6 years of age with schistosomiasis: research and development of a pediatric formulation of praziquantel

**DOI:** 10.1186/s40249-017-0336-9

**Published:** 2017-08-03

**Authors:** Jutta Reinhard-Rupp, Katharina Klohe

**Affiliations:** 1 Merck Coinsins, Coinsins, Switzerland; 2 Global Schistosomiasis Alliance, Westenriederstrasse 10, 80331 Munich, Germany

**Keywords:** Schistosomiasis, Pediatric formulation, Children, Praziquantel

## Abstract

Schistosomiasis is a parasitic disease caused by blood flukes. The disease is caused by an inflammatory reaction to parasite eggs retained in the liver, bladder and reproductive organs. According to 2017 World Health Organization (WHO) estimates 220 million people are potentially infected, from which probably 10% are children under 6 years of age. The regular treatment approach of a single, oral dose of 40 mg/kg body weight with praziquantel however, is difficult for children under the age of 6, leaving them without a treatment option.

In order to address this important gap in treatment target populations, an international public-private partnership that works on a not-for-profit basis in the field of drug research and development for schistosomiasis was established in 2012. This is called the Pediatric Praziquantel Consortium. Its mission was and continues to be to develop, register and provide access to a suitable pediatric praziquantel formulation for treating schistosomiasis in preschool-age children (3–6 months up to 6 years).

The Target Product Profile for the pediatric formulation of praziquantel that would be suitable to treat children as young as 3–6 months was then defined by a group of experts, including members from the Pediatric Praziquantel Consortium partner organizations as well as experts from WHO (as observer) and schistosomiasis endemic countries.

The development of the drug is ongoing and the Pediatric Praziquantel Consortium aims to submit the regulatory dossier for marketing approval in endemic countries and WHO prequalification in 2018/19 with approval and product launch for schistosomiasis pediatric case management in key endemic countries in 2019. Ultimately, the goal is for the product to be considered for a large-scale mass distribution program by 2022.

**Electronic supplementary material:**

The online version of this article (doi:10.1186/s40249-017-0336-9) contains supplementary material, which is available to authorized users.

## Multilingual abstract

Please see Additional file 1 for translations of abstract into six official working languages of the United Nations.

## Background

Schistosomiasis is a parasitic disease caused by blood flukes. The main species infecting humans are *Schistosoma mansoni* and *S. haematobium*. The disease is caused by an inflammatory reaction to parasite eggs retained in, amongst others, liver, bladder and reproductive organs [1]. Accumulation of heavy infections with time leads to severe manifestations of schistosomiasis. Children, if exposed to infection can develop morbidity and mortality early in life. According to 2017 World Health Organization estimates 220 million people are potentially infected, from which probably 10% are children under 6 years of age. The approach to control schistosomiasis is based on regular treatment with a single, oral dose of 40 mg/kg body weight with praziquantel [2]. The main target population for treatment are children of school age (6 to 14 years of age reached via large-scale school-based campaigns) [3, 4]. This approach has recently highlighted the diagnosis and treatment needs of preschool-age children [5–7], particularly given that praziquantel is safe, well-tolerated and efficacious [8, 9]. However, the available 600 mg praziquantel tablet is difficult for the younger population. No clinical data of an accurate dose are available, and it requires crushing the tablets, resulting in inaccuracy of dosing [10, 11]. Moreover, the current praziquantel product unveils a bitter taste to which infants and children react unfavourably, leading to compliance issues [12].

## Addressing the therapeutic gap: the pediatric Praziquantel consortium

Recognizing the medical need of schistosome infected preschool-age children including infants and toddlers [13], an international public-private partnership that works on a not-for-profit basis in the field of drug research and development for schistosomiasis was established in 2012. Its mission is to develop, register and provide access to a suitable pediatric praziquantel formulation for treating schistosomiasis in preschool-age children (3–6 months up to 6 years). The Consortium operates through engaging some of the best science and most experienced public and private partners in pharmaceutical product research and development. It now consists of seven partners, each of them contributing through funding, expertise and resources (in-kind contribution) to the program: Merck (Germany), Lygature (The Netherlands), Astellas Pharma Inc. (Japan), Swiss TPH (Switzerland), Farmanguinhos (Brazil), Simcyp (UK) and SCI (UK). The partners have formed a core project team, led by a Merck project leader, that is responsible for the overall management of the development program (www.pediatricpraziquantelconsortium.org). The Consortium is steered by a Board which consists of top management representatives of the partners. As independent coordinator, Lygature provides governance to the consortium in terms of progress, finance, IP management and collaborations.

The Consortium partners have successfully secured grants from the Bill and Melinda Gates Foundation in 2013, and from the Global Health Innovative Technology (GHIT) Fund in 2014, 2015 and 2016. Beyond the partners contributing expertise and resources, the Consortium consults international academics, experts from endemic countries, funding agencies, the WHO and non-governmental organizations via expert meetings [14].

## Pediatric Praziquantel: target product profile

In March 2012 a group of experts, including members from the consortium partner organizations as well as experts from WHO (as observer) and schistosomiasis endemic countries defined the Target Product Profile for a pediatric formulation of praziquantel that would be suitable to treat children as young as 3–6 months (Table 1).

**Table 1:** Target Product Profile for pediatric praziquantel

Description	Praziquantel pediatric^a^
Indication	Treatment of schistosomiasis (*Schistosoma mansoni and*. H*aematobium*)
Target population	Children (3–6 months to 6 years) with proven schistosomiasis infection able to take oral medication and not receiving co medication for other diseases.
Dosage and administration	Orally disintegrating tablet (taste masked) administered orally (as intact tablet or dissolved in water) as a single dose treatment (in mg/kg of body weight).
Target efficacy	Minimum case scenario: Cure rate in phase III trial is between 60-75% Base case scenario: Cure rate in phase III trial is 75% High case scenario: Cure rate in phase III trial is above 75%
Target safety	A safety and tolerability profile equal or better than that of current praziquantel tablets.
Stability in WHO zone IVB climatic conditions (hot, humid climate, 30 °C/75% RH)	Minimum case scenario: stable for 18–24 months Base case scenario: Stable for 24–36 months. High case scenario: Stable for >36 months
Packaging	Primary packaging: HDPE bottles with or without desiccant (low bulk weight and volume packaging material) if feasible. Package sizes that allow optimal use under public health program conditions. Approx. 50–100 units per bottle
Key statement	The new formulation will be suitable for pediatric use in Sub-Saharan Africa, Brazil and other endemic countries. It will be appropriate for use in both case management administration and community directed mass treatment (i.e. large-scale preventive chemotherapy). This will require further post regulatory approval field studies to assess effectiveness.

^a^The current development plan is evaluating the L-Praziquantel and the racemic mixture

## Research & Development: Strategic approach

**Fig. 1:**
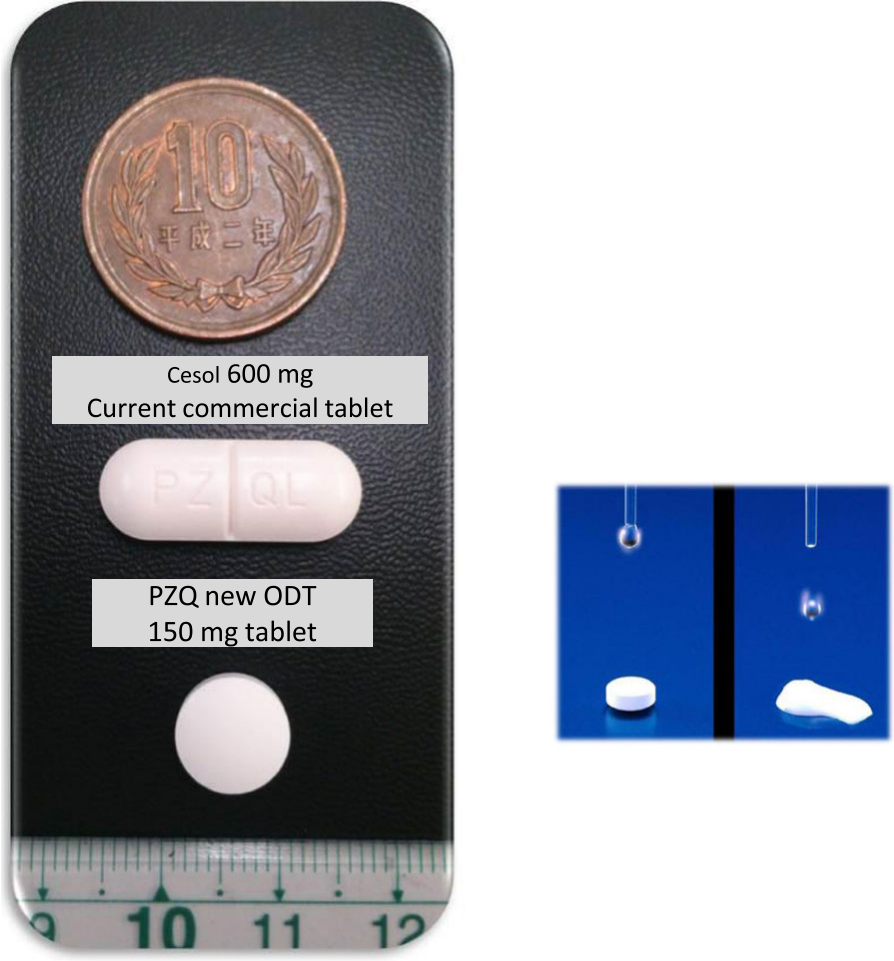
Comparison of the original vs. the pediatric Praziquantel formulation

To optimize chances of success, the Consortium is developing an innovative child-friendly orodispersible formulation with improved palatability (orodispersible (150 mg tablets), for the L-praziquantel (L-PZQ) – devoid of the biologically inactive D-PZQ enantiomer as well as for the praziquantel racemate mixture. Research suggests the usefulness of 150 mg tablet sizes, allowing adequate dosing for children of different body weights between 6 months and up to 6 years of age [15]. The study hypothesis for the development of the L-PZQ development is that it could result in a reduced dosage per treatment as compared to racemate and with better tolerability and less side effects [16] (Fig. 1).

The drug development program consists of two major parts: preclinical development, and clinical development guided and coordinated through the consortium core project team.

1. Preclinical development consists of major activities aimed at developing and providing the research material for the clinical evaluation. The activities include: manufacture of the active pharmaceutical ingredient (API), undertaken by Merck. Development and manufacture of novel orally dispersible tablet (ODT) formulation candidates, undertaken by Astellas, Merck and Farmanguinhos. Development and validation of analytical and bioanalytical methods by Astellas, Merck and Farmanguinhos. Metabolism, pharmacokinetics toxicology and PK modeling studies by Merck, Swiss TPH and Simcyp. These activities are conducted under the framework of Good Manufacturing Practice (GMP) and Good Laboratory Practices l– according to regulatory authority requirements.
2. Clinical development: Our clinical development program is in line with USA Food and Drug Administration recommendations for pediatric development. The relevant studies have been designed with the input of clinical experts from endemic countries and have been discussed with representatives from endemic country regulatory authorities. The program comprises several studies:
  -  Phase I bioavailability studies in healthy adult volunteers in South Africa, to determine the pharmacokinetic properties of the Racemate (Rac)-PZQ and L-PZQ formulation candidates in comparison to the current 600 mg PZQ commercial racemate tablet formulation (Cesol). These studies were completed in 2015.
  -  A taste study in Tanzanian school-age children consisting of 5 groups cross-over randomized study in children age 6–11 years (*n* = 48), to assess the taste and the overall palatability of the new candidate pediatric ODT formulations versus the current drug (600 mg). This study was completed in 2015.
  -  Phase II PK/PD dose finding study with L-PZQ and rac-PZQ ODTs + control commercial PZQ (in Côte d’Ivoire) consisting of part 1 that includes children age 2–6 years infected with *S. mansoni*, followed by a patient group of younger children age 6 months-2 years infected with *S. mansoni*. A planned part 2 will include children age 2–6 years infected with *S. haematobium*.
  -  Phase III study with either L-PZQ or rac-PZQ ODTs to demonstrate efficacy/safety of PZQ ODTs in preschool-age children, expected to start in 2017.

All the clinical trials are conducted according to Good Clinical Practice and applicable ethical guidelines and regulations, in order to protect the wellbeing and rights of the adults and children enrolled in the studies. The clinical trial program is designed to reflect public health needs and priorities of countries endemic for schistosomiasis.

## Future outlook

The Pediatric Praziquantel Consortium aims to submit the regulatory dossier for marketing approval in endemic countries and WHO prequalification in 2018/19 with approval and product launch for schistosomiasis pediatric case management in key endemic countries in 2019. This will allow the safety data base to be built as well as to establish the product effectiveness under field conditions with the scope that by 2022 the product could be considered for a large-scale mass distribution program for morbidity control. In parallel, the Consortium will continue to explore diverse financial mechanisms that could guarantee a sustainable access to those in need.

## Conclusion

The Pediatric Praziquantel Consortium is an international public-private partnership that was established in 2012 in recognition of the medical needs of schistosome infected preschool-age children, including infants and toddlers. If the prevalence and intensity levels of schistosomiasis and the associated physiological damages this disease can cause are to be addressed in an all-inclusive manner, it is imperative that a treatment form for children below the age of 6 is found. The work of the Pediatric Consortium and their research and development plan should ensure that the treatment of pre-school-aged children can become a reality in the near future. It is hoped that treating this population group will help make major progress towards the elimination of schistosomiasis through these to ensure access to treatment.

## Additional file

Additional file 1: Multilingual abstract in the five official working languages of the United Nations. (PDF 585 kb)

## Abbreviations

PZQ: Praziquantel
SCI: Schistosomiasis Control Initiative
WHO: World Health Organization
