# Supplementary material for: Developing a comprehensive response for treatment of children under 6 years of age with schistosomiasis: research and development of a pediatric formulation of praziquantel
Source: Infect Dis Poverty. 2017 Aug 3;6:122. doi: 10.1186/s40249-017-0336-9 (PMC5541653; doi:10.1186/s40249-017-0336-9)
Supplement: Additional file 1: — Multilingual abstract in the five official working languages of the United Nations. (PDF 585 kb) [file 40249_2017_336_MOESM1_ESM.pdf]

Translation of the abstract into the six official working languages of the United Nations

تطوير استجابة شاملة لعلاج الأطفال دون سن 6 سنوات من البلهارسيا: البحث والتطوير لتركيبات الأطفال من مستحضر برازيكوانتيل (praziquantel)

Jutta Reinhard-Rupp, Katharina Klohe

المستخلص

داء البلهارسيا مرض طفيلي تسببه مثقبات الدم (blood flukes). ويرجع هذا المرض عن طريق رد فعل الالتهابات مع بيض الطفيلي المحتفظ بها في الكبد والمثانة والأعضاء التناسلية. ووفقا لمنظمة الصحة العالمية لعام 2017، تشير تقديرات منظمة الصحة العالمية إلى احتمال إصابة 220 مليون شخص بالعدوى، ومن المحتمل أن 10٪ منهم من الأطفال دون سن السادسة. ومع ذلك، فإن أسلوب العلاج المنتظم لجرعة واحدة، عن طريق الفم تبلغ 40 ملغم / كغم من وزن الجسم مع البرازيكوانتيل، يصعب على الأطفال دون سن 6 سنوات، مما يجعلهم دون خيار اخر للعلاج

ومن أجل معالجة هذه الفجوة الهامة في السكان المستهدفين للعلاج، أنشئت في عام 2012 شراكة دولية بين القطاعين العام والخاص تعمل على أساس غير هادف للربح في مجال البحث والتطوير في مجال الادوية من أجل داء البلهارسيا. ويطلق على هذه الشراكة اسم اتحاد برازيكوانتيل للأطفال. وكانت مهمتها وما زالت هي تطوير وتسجيل وتوفير إمكانية الوصول إلى تركيبة مناسبة من البرازيكوانتيل للأطفال لعلاج البلهارسيا في الأطفال في سن ما قبل المدرسة (3-6 أشهر حتى 6 سنوات).

ومن ثم تم تعريف ملف المنتج المستهدف لتركيبات الأطفال من برازيكوانتيل التي من شأنها أن تكون مناسبة لعلاج الأطفال الذين تتراوح أعمارهم بين 3-6 أشهر من قبل مجموعة من الخبراء، بما في ذلك تحالف أعضاء من المنظمات المشتركة فى برازيكوانتيل للأطفال وكذلك خبراء من منظمة الصحة العالمية (كمراقبين) والبلدان المتوطنة بالبلهارسيا.

تطوير الادوية مستمر ويهدف اتحاد برازيكوانتيل للأطفال لتقديم الملف التنظيمي للموافقة التسويقية في البلدان المتوطنة ومنظمة الصحة العالمية للتأهيل في 19/2018 مع الموافقة وإطلاق المنتج لإدارة حالات الأطفال المصابين بالبلهارسيا في البلدان المتوطنة الرئيسية في 2019 في نهاية المطاف، والهدف من ذلك هو رؤية للمنتج في برنامج شامل للتوزيع على نطاق واسع بحلول عام 2022.

Translated from English version into Arabic by Khaled Zayed

制定 6 岁以下儿童血吸虫病的综合治疗方案：吡喹酮儿科剂型的研发

Jutta Reinhard-Rupp, Katharina Klohe

摘要

血吸虫病是一种寄生虫病，由血吸虫引起。该病是由人体对肝脏、膀胱和生殖器官中血吸虫虫卵的炎症反应引起的。据 WHO 估计，2017 年有 2.2 亿人感染血吸虫，其中 10%是 6 岁以下儿童。血吸虫病常规疗法是按 40 mg/kg 体重剂量单次口服吡喹酮，但是在 6 岁以下儿童存在挑战，因此他们没有合适的治疗方案可供选择。

为解决这一目标人群治疗的重大差距，2012 年建立了一个全球的公私合作伙伴关系，即儿童吡喹酮协作组，致力于非盈利性质的针对血吸虫病的开展药物研发。其任务是研究、登记和提供合适的儿科剂型吡喹酮，以治疗学龄前儿童（3-6 个月到 6 岁间）的血吸虫病。

来自于儿童吡喹酮协作组和 WHO 的专家（后者为观察员）以及血吸虫病流行国家的专家对适合 3-6 个月儿童的儿科剂型吡喹酮的目标产品特征进行了界定。

该项药物研发正在进行中。儿童吡喹酮协作组的目标是 2018/2019 年在流行国家提交上市认证的监管档案并获得 WHO 的预认证；2019 年在主要流行国家进行血吸虫病儿科病例管理的产品启动。最终目标是考虑在 2022 年将产品进行大规模的分配。

Translated from English version into Chinese by Men-Bao Qian, edited by Pin Yang

CHINESE CENTER FOR DISEASE CONTROL AND PREVENTION
NATIONAL INSTITUTE OF PARASITIC DISEASES

Elaboration d'une action globale pour le traitement des enfants de moins de 6 ans atteints de la schistosomiase: recherche et développement d'une formulation pédiatrique du praziquantel

Jutta Reinhard-Rupp, Katharina Klohe

Résumé

La schistosomiase est une maladie parasitaire causée par les douves du sang. La maladie est due à une réaction inflammatoire aux œufs du parasiste conservés dans le foie, la vessie et les organes reproducteurs. D'après les estimations de l'Organisation Mondiale de la Santé (OMS) en 2017, 220 millions de personnes pourraient être infectées, dont 10% sont des enfants de moins de 6 ans. L'approche du traitement ordinaire d'une dose orale unique de 40 mg/kg de poids corporel avec le praziquantel est toutefois difficile pour les enfants de moins de 6 ans, les laissant ainsi à aucune une option de traitement.

Afin de remédier à cette lacune importante dans le traitement des populations cibles, un partenariat public - privé international, à but non lucratif, dans le domaine de la recherche et du développement de médicaments contre la bilharziose a été créé en 2012. Il s'agit du Consortium pour une formulation Pédiatrique du Praziquantel. Sa mission a été et continue d'être élaborée, d'enregistrer et de fournir l'accès à une formulation appropriée pédiatrique du praziquantel pour le traitement de la schistosomiase chez des enfants d'âge préscolaire (3-6 mois à 6 ans).

Le Profil du Produit Cible pour la formulation pédiatrique du praziquantel qui serait approprié pour traiter des enfants aussi jeunes qu'à l'âge de 3-6 mois a été ainsi défini par un groupe d'experts, y compris les membres des organismes partenaires du Consortium, ainsi que des experts de l'OMS (en qualité d'observateur) et des pays où la schistosomiase est endémique.

Le développement du médicament est en cours et le Consortium pour une formulation Pédiatrique du Praziquantel vise à présenter le dossier de réglementation pour approbation de commercialisation dans les pays endémiques, et pour certification préalable de l'OMS en 2018/19, avec approbation et lancement de produit pour le traitement des cas pédiatriques de la schistosomiase dans les principaux pays endémiques en 2019. En bout de ligne, l'objectif est d'arriver à ce que le produit soit considéré pour un programme de distribution de masse à grande échelle en 2022.

Translated from English version into French by Kokouvi Kassegne

Разработки комплексных мер для лечения детей в возрасте до 6 лет с шистосомозом: Научные исследования и разработки по педиатрическимрецептурам празиквантела

Jutta Reinhard-Rupp, Katharina Klohe

Введение

Шистосомоз-это паразитарное заболевание, вызываемое сосальщиками крови. Болезнь вызвана воспалительной реакцией на яйца паразитов сохраняются в печени, мочевом пузыре и репродуктивных органах. По данным Всемирной организации здравоохранения (ВОЗ) 2017 -го года около 220 миллионов человек потенциально инфицированны, из которых примерно 10% составляют дети в возрасте до 6 лет. Регулярный подход к лечению одного, внутрь в дозе 40 мг/кг массы тела с празиквантелом, однако, это трудно для детей, которые в возрасте до 6 лет, нет варианта лечения для них.
Для того, чтобы решить этот важный пробел в цели лечения населения, Международное государственно-частное партнерство было создано в 2012 году, которое действует на некоммерческой основе в области исследования и разработки препаратов для шистосомоза. Это называется "Консорциум детского Празиквантела". Его миссия была и остается разработкой, регистрацией и обеспечить доступ к соответствующей разработке детского празиквантела для лечения шистосомоза у детей дошкольного возраста (3-6 месяцев до 6 лет).
"Целевой профиль продукта" для разработки детского празиквантела, подходящий для лечения детей в возрасте 3-6 месяцев и затем был определен группой экспертов, в том числе члены организаций-партнеров консорциума детского Празиквантела, а также эксперты ВОЗ (в качестве наблюдателя) и из эндемичных стран по шистосомозу.
Разработка препарата ведется и консорциум детского Празиквантела стремится представить регулируемое досье для получения разрешения на продажу в эндемичных странах и на предварительную квалификацию ВОЗ в 2018/19 с утверждением и реализацией продукции для управления дел педиатрического шистосомоза в ключевых эндемичных странах в 2019 году. В конечном счете, цель для продукта состоит в том, чтобы продукты были рассмотрены в течение широкомасштабной программы массовой рассылки к 2022 году.

Translated from English version into Russian by Hao-Qi Zhang

Desarrollo de una respuesta integral para el tratamiento de niños menores de 6 años con esquistosomiasis: Investigación y desarrollo de una formulación pediátrica de praziquantel

Jutta Reinhard-Rupp, Katharina Klohe

Abstracto

La esquistosomiasis es una enfermedad parasitaria causada por colirios sanguíneos. La enfermedad es causada por una reacción inflamatoria a los huevos del parásito retenidos en el hígado, la vejiga y los órganos reproductivos. Según la Organización Mundial de la Salud (OMS) en el 2017 estima que 220 millones de personas están potencialmente infectadas, de las cuales probablemente el 10% son niños menores de 6 años de edad. El tratamiento regular de una única dosis oral de 40 mg / kg de peso corporal con praziquantel, sin embargo, es difícil para los niños menores de 6 años, dejándolos sin

opción de tratamiento. Con el fin de abordar esta importante brecha en las poblaciones objetivo de tratamiento, en 2012 se estableció una asociación internacional público-privada que trabaja sin ánimo de lucro en el campo de la investigación y el desarrollo de medicamentos para la esquistosomiasis. Esto se llama el Consorcio Praziquantel Pediátrico. Su misión era y sigue siendo desarrollar, registrar y proporcionar acceso a una formulación praziquantel pediátrica adecuada para el tratamiento de la esquistosomiasis en niños en edad preescolar (3-6 meses hasta 6 años). El perfil del producto objetivo para la formulación pediátrica de praziquantel que sería adecuado para tratar a los niños de tan sólo 3-6 meses fue definido por un grupo de expertos, incluidos miembros de las organizaciones asociadas del Consorcio Pediátrico Praziquantel, así como expertos de la OMS (como observador) y países de esquistosomiasis endémica. El desarrollo del fármaco está en curso y el Consorcio Pediátrico Praziquantel pretende presentar el expediente regulador para la aprobación de la comercialización en los países endémicos y la precalificación de la OMS en 2018/19 con la aprobación y el lanzamiento del producto para la gestión pediátrica de la esquistosomiasis en países endémicos clave en 2019. Finalmente, el objetivo es que el producto sea considerado para un programa de distribución masiva a gran escala para 2022.

Translated from English version into Spanish by Laura C Vicente Rodriguez
